# Factors associated with immunization against hepatitis B among primary health care professionals

**DOI:** 10.47626/1679-4435-2022-790

**Published:** 2023-02-13

**Authors:** Gabrielly Alves Mota, Lara Vívian Paixão Fernandes, Luana Alkmim Fonseca, Maria Izabel de Azevedo Ferreira, Vitor Lucas Bonfim Mendes, Carlos Roberto Santos Lima, Anamaria de Souza Cardoso

**Affiliations:** 1 Medicina, Centro Universitário FIPMoc, Montes Claros, MG, Brazil; 2 Enfermagem, Universidade Estadual de Montes Claros, Montes Claros, MG, Brazil

**Keywords:** Hepatitis B, immunization, health personnel, serologic tests, Hepatite B, imunização, pessoal de saúde, testes sorológicos

## Abstract

**Introduction:**

The hepatitis B virus is the cause of one of the major public health problems worldwide. The infection may affect the entire population equally; however, health care professionals are part of a group that is more vulnerable to the disease, since they are exposed to both occupational and daily hazards.

**Objectives:**

To investigate the prevalence and factors associated with the immunization of health care professionals against the hepatitis virus type B, in the city of Montes Claros, state of Minas Gerais, Brazil.

**Methods:**

This was a cross-sectional and quantitative study, conducted with primary health care professionals. Using a random cluster sampling, 209 medical professionals, nurses, and nursing technicians who were interested in participating in the research were selected. A structured questionnaire was applied, and blood sampling was performed for the analysis of hepatitis B surface antibody titers. Finally, a descriptive and bivariate statistical analysis was conducted.

**Results:**

Data have shown that 91.8% of the professionals had complete immunization against the hepatitis B virus, that is, they had taken the three recommended doses of the vaccine. However, 13.9% of the sample, even after vaccination, was non-reactive (titers < 10 IU/mL hepatitis B surface antibody). Most of the professionals (94.3%) had direct contact with needlesticks/sharps at work and none of the participants reported a previous infection by the virus.

**Conclusions:**

Although most participants had complete immunization, the total result of individuals who did not obtain seroconversion was eminent, so the importance of the hepatitis B surface antibody test must be disseminated in the context of public health.

## Abstract (pt)

**Introdução:**

O vírus da hepatite B é o causador de um dos grandes problemas de saúde pública mundial. A infecção pode acometer toda a população de igual maneira; entretanto, os profissionais de saúde são parte de um grupo mais vulnerável à doença, visto que são expostos tanto ao risco ocupacional quanto ao risco cotidiano.

**Objetivos:**

Investigar a prevalência e os fatores associados à imunização de profissionais de saúde contra o vírus da hepatite viral tipo B na cidade de Montes Claros, Minas Gerais, Brasil.

**Métodos:**

Tratou-se de um estudo transversal e quantitativo, realizado com profissionais de saúde da atenção primária. Por amostragem aleatória por conglomerado, foram selecionados 209 profissionais médicos, enfermeiros e técnicos de enfermagem que tiveram interesse em participar da pesquisa. Foi aplicado um questionário estruturado, e foi realizada coleta de sangue para análise de títulos de anticorpo contra o antígeno de superfície da hepatite B. Por fim, a análise estatística descritiva e bivariada foi efetuada.

**Resultados:**

Os dados evidenciaram que 91,8% dos profissionais tinham imunização completa contra o vírus da hepatite B - ou seja, haviam tomado as três doses preconizadas da vacina. Entretanto, 13,9% da amostra, mesmo após a vacinação, se mostrou não reagente (títulos < 10 UI/mL de anticorpo contra o antígeno de superfície da hepatite B). A maioria (94,3%) dos profissionais tinha contato direto no trabalho com materiais perfurocortantes, e nenhum participante relatou infecção prévia pelo vírus.

**Conclusões:**

Apesar do fato de que a maioria dos participantes tinha imunização completa, o resultado total de indivíduos que não obtiveram soroconversão foi eminente, de modo que há de se divulgar a valia do exame de anticorpo contra o antígeno de superfície da hepatite B no contexto de saúde pública.

## INTRODUCTION

Viral hepatitis is a disease caused by etiological agents of universal distribution, which have hepatotropism in common but differ in transmission routes and subsequent complications.^1^ Hepatitis B, in particular, is a disease caused by infection with the hepatitis B virus (HBV), belonging to the Hepadnaviridae family, of which humans are the natural host.^2^

Public health authorities have ranked the reduction in vaccine-preventable diseases as one of the top 10 public health achievements of the 21st century.^3^ The hepatitis B vaccine is an integral part of this achievement and is the most effective means of preventing active HBV infection and its consequences.^4^

This virus can be transmitted parenterally, vertically, or sexually, and hepatitis B is considered a sexually transmitted infection (STI).^5^ The greatest potential for infection by the virus is when it is present in serum and semen. Therefore, individuals with multiple sexual partners, who use injection drugs, and those who interact with people in the risk group are highly susceptible to contamination.^6^

Besides the usual risk, health care professionals are one of the main risk groups for HBV infection because they are in continuous and prolonged contact with infectious materials and often need to handle sharp objects in patients affected by the virus.^7^ The procedures that most offer risk to the professional are also those required recurrently in hospital or outpatient settings, such as venipuncture, drug dilution, blood glucose testing, and intramuscular drug administration.^8^

Hepatitis B is a disease of great social impact because it has a wide spectrum of contamination and it may be asymptomatic, but it can also have a more severe form, become chronic, and even evolve to complications, generating high morbidity and mortality in the population.^9^ Moreover, because HBV is a DNA virus, 10 to 20% of inactive carriers may have spontaneous reactivation of HBV replication and exacerbations of hepatitis after years of quiescence. This reactivation is characterized by the reappearance of replication markers and active necroinflammatory liver disease.^10^

In order to decrease the transmission and reduce the incidence of severe cases of the disease, the hepatitis B vaccine was introduced at the beginning of 2000 by the Brazilian National Immunization Program (NIP).^11^ The vaccine should be administered as early as possible to all Brazilians in a four-dose schedule, at 0, 1, 2, and 6 months after birth, in unvaccinated people, in the main risk groups, or people with an incomplete vaccination schedule.^5^

Even with the availability of the vaccine by the Brazilian Unified Health System (SUS), many people in Brazil do not have a complete vaccination card, due to factors such as lack of awareness of the risk of infection and lack of information about the transmission and severity of the disease.^12^ Furthermore, there is still the possibility that even with a complete vaccination schedule, some individuals may not seroconvert.^13^

Thus, for an effective immunization, the post-vaccine serological test is indicated for risk groups, especially health professionals, and, in cases of negative serological titers, revaccination with one dose and serological repetition is recommended.^5^ Thus, non-immunization is an important risk factor that, associated with the other socioeconomic barriers present in Brazil, still makes hepatitis B an issue of great epidemiological importance. Thus, the present study aimed to investigate the prevalence and factors associated with the immunization of health professionals against HBV in the municipality of Montes Claros, state of Minas Gerais (MG), Brazil.

## METHODS

### STUDY DESIGN

This was a quantitative, cross-sectional, descriptive study with an analytical stage comprising health professionals from primary care in the municipality of Montes Claros (MG, Brazil), conducted between August 2020 and January 2021. According to data from the Brazilian National Registry of Health Establishments (Cadastro Nacional de Estabelecimentos de Saúde - CNESNet), in this period, Montes Claros had 86 Basic Health Units, which included 142 family health teams.

### SAMPLING AND DATA COLLECTION PROCEDURES

The sample size was calculated using the single population proportion formula, using a 50% endpoint, 95% confidence level, and 5% margin of error. For the total population of 428 healthcare professionals, the sample size was 209 healthcare professionals.

To select the individuals who would join in the research, random sampling by conglomerate was used. Thus, 86 health units were listed and then drawn. The first 54 health units drawn were included in the research, completing the number of 209 participating professionals.

Physicians, nurses, and nursing technicians were included in the survey. Exclusion criteria included the impossibility of blood sample collection, contaminated blood samples, or inadequate questionnaire response.

In the first phase of the study, an educational lecture was given to the health professionals of the health units included in the study. The researchers explained the importance of hepatitis B vaccination and hepatitis B surface antigen (anti-Hbs) antibody testing in the healthcare setting to the health care teams at these sites. The participants were then invited to participate in the survey. This procedure was repeated in the healthcare units until the number of 209 professionals needed for the study was reached.

Data collection was performed by the researchers in collaboration with a qualified nurse. A structured questionnaire was applied to the health professionals who volunteered for the research. Next, blood samples were collected by peripheral venipuncture using a vacuum system to perform the anti-Hbs test. The questionnaire included sociodemographic and work information, and the immunization status of the individuals. The methodology used in the serological test was chemiluminescence, with the result expressed as a unit value, in which individuals with titers ≥ 10 IU/mL of anti-Hbs in the blood were classified as reagent or immune to HBV infection.

### STUDY VARIABLES

The anti-Hbs titer and vaccination status of the sample were the dependent variables in this study. The independent variables were defined from a theoretical model that was based on reference studies in the field.

The sociodemographic data, work characteristics, and immunization status of the study participants were surveyed. Age, time of the last vaccine dose, and the number of times individuals had previous accidents with biological materials were recorded based on the respondents’ answers. The occupation was classified according to the health occupations included in the study (physician, nurse, or nursing technician). Other adjustment variables used were sex (female, male), skin color/race (black, brown, yellow, white, indigenous), work institution (outpatient clinic, Family Health Strategy [FHS], hospital, Mobile Emergency Care Service [SAMU]), time of work in the institutions reported (< 5 years, 5-10 years, > 10 years), and direct contact in the work environment (perforating-cutting materials, minimally invasive procedures, surgical procedures, biological materials in laboratories). Prior HBV infection and prior knowledge of anti-Hbs testing were treated as dichotomous variables (yes or no).

### DATA ANALYSIS

Descriptive analyses were performed for all variables, and the calculations of relative frequencies were presented in table form. For the bivariate analyses, Pearson’s chi-square test was used, establishing p values lower than 0.05 as significant. Data analysis was conducted in the SPSS, version 20.

### ETHICAL CONSIDERATIONS

According to the standards of Resolution 510/2016 of the National Health Council (NHC), the study was approved by the Research Ethics Committee of the FIPMoc Centro Universitário (UNIFIPMoc) (no. 4,243,379), under number 36794520.8.0000.5109. Confidentiality and secrecy of information were ensured, and written informed consent was obtained from all participants. In addition, authorization to conduct the research was obtained from the Municipal Health Department of the municipality of Montes Claros, MG, Brazil.

## RESULTS

The sample consisted of 209 health care professionals and was composed predominantly of women (80.4%). The mean age was 37 years (standard deviation [SD] = 8.7; range 20-72), and most participants (39.7%) were between 30 and 39 years. Regarding skin color/race, 62.7% declared themselves to be brown. Of the participants, 37.8% were nursing technicians. All participants were FHS workers, of which 42.1% had less than 5 years of experience, 94.3% had direct contact with perforating-cutting materials, and 69.9% had never experienced accidents with biological materials (Table 1).

**Table 1:** Sociodemographic and work characteristics of primary health care professionals in Montes Claros, state of Minas Gerais, Brazil, 2020

Characteristics	Frequency (n)	Percentage (%)
Sex		
Women	168	80.4
Men	41	19.6
Age (years)		
20-29	46	22.0
30-39	83	39.7
40-49	61	29.2
50-59	15	7.2
≥ 60	4	1.9
Self-declared color/race		
Black	14	6.7
Brown	131	62.7
Yellow	9	4.3
White	55	26.3
Profession		
Physician	57	27.3
Nurse	73	34.9
Nursing technician	79	37.8
Institution of work		
Outpatient clinic	3	1.4
Family health strategy	209	100.0
Hospital	18	8.6
Mobile emergency care service	3	1.4
Time of current work experience (years)		
< 5	88	42.1
5-10	64	30.6
> 10	57	27.3
Direct contact in the work environment with:		
Perforating materials	197	94.3
Minimally invasive procedures	109	52.2
Surgical procedures	12	5.7
Biological materials in laboratories	7	3.3
Number of accidents with biological materials in the workplace		
0	146	69.9
1	41	19.6
2	18	8.6
≥ 3	4	1.9

A large proportion of the healthcare workers (91.8%) were immunized with more than three doses of the HBV vaccine. Of the workers tested, 40.2% had taken the last dose of the vaccine within 2 to 5 years, and none reported previous HBV infection. Regarding the anti-Hbs test, 13.9% were non-reactive. It was found that 72.2% of the participants had been previously tested (Table 2).

**Table 2:** Immunological and serological status regarding hepatitis B virus (HBV) in primary care professionals in Montes Claros, state of Minas Gerais, Brazil, 2020

Characteristics	Frequency (n)	Percentage (%)
HBV immunization (doses)		
1	6	2.9
2	11	5.3
≥ 3	192	91.8
Time of last vaccine dose (years)		
≤ 1	23	11.0
2-5	84	40.2
6-10	55	26.3
11-15	23	11.0
> 15	24	11.5
Anti-Hbs tests		
Non-reactive	29	13.9
Reactive	180	86.1
Previous knowledge of the anti-Hbs test		
Yes and already took it	151	72.2
Yes, knew its importance, but did not take it	54	25.8
Yes, but did not know its importance and did not take it	1	0.5
No previous knowledge	3	1.4

Anti-Hbs = antibody to the surface antigen of hepatitis B.

Among the non-reactive professionals, 15 of them had taken the last dose of the vaccine more than 5 years ago, which corresponded to 7.2% of the sample studied. The age of workers was the only variable that showed statistical significance when associated with the number of doses taken by health professionals (p ≤ 0.001) and seroconversion after vaccination (p = 0.013). No association was observed between the negative anti-Hbs test and the other independent variables, which may be associated with the small sample size (Tables 3 and 4).

**Table 3:** Factors associated with hepatitis B virus vaccination status in primary care professionals in Montes Claros, state of Minas Gerais, Brazil, 2020

Characteristics	1 dose n (%)	2 doses n (%)	≥ 3 doses n (%)	p^*^
Sex				0.544
Women	4 (2.4)	8 (4.8)	156 (92.9)	
Men	2 (4.9)	3 (7.3)	36 (87.8)	
Age (years)				0.000
20-29	2 (4,3)	3 (6.5)	41 (89.1)	
30-39	2 (2.4)	3 (3.6)	78 (89.1)	
40-49	2 (3.3)	2 (3.3)	57 (93.4)	
50-59	0 (0.0)	0 (0.0)	15 (100.0)	
≥ 60	0 (0.0)	3 (75.0)	1 (25.0)	
Self-declared color/race/				0.533
Black	0 (0.0)	1 (7.1)	13 (92.9)	
Brown	3 (2.3)	5 (3.8)	123 (93.9)	
Yellow	0 (0.0)	0 (0.0)	9 (100.0)	
White	3 (5.5)	5 (9.1)	47 (85.5)	
Profession				0.189
Physician	3 (5.3)	3 (5.3)	51 (89.5)	
Nurse	1 (1.4)	1 (1.4)	71 (97.3)	
Nursing technician	2 (2.5)	7 (8.9)	70 (88.6)	
Work institution				-
Outpatient clinic	0 (0.0)	0 (0.0)	3 (100.0)	
Family health strategy	6 (2.9)	11 (5.3)	192 (91.9)	
Hospital	0 (0.0)	2 (11.1)	16 (88.9)	
Mobile emergency care service	0 (0.0)	0 (0.0)	3 (100.0)	
Time of work experience (years)				0.191
< 5	3 (3.4)	6 (6.8)	79 (89.8)	
5-10	1 (1.6)	0 (0.0)	63 (98.4)	
> 10	2 (3.5)	5 (8.8)	50 (87.7)	
Direct contact with:				-
Perforating materials	6 (3.0)	10 (5.1)	181 (91.9)	
Minimally invasive procedures	2 (1.8)	5 (4.6)	102 (93.6)	
Surgical procedures	0 (0.0)	0 (0.0)	12 (100.0)	
Biological materials in laboratories	0 (0.0)	0 (0.0)	7 (100.0)	
Number of previous accidents with biological materials				0.925
0	4 (2.7)	7 (4.8)	135 (92.5)	
1	2 (4.9)	3 (7.3)	36 (87.8)	
2	0 (0.0)	1 (5.6)	17 (94.4)	
≥ 3	0 (0.0)	0 (0.0)	4 (100.0)	

* Pearson’s chi-square test.

**Table 4:** Factors associated with seroconversion after hepatitis B vaccination in primary care health professionals in Montes Claros, state of Minas Gerais, Brazil, 2020

Characteristics	Non-reactive Anti-Hbs	Reactive Anti-Hbs	p^*^
Sex			0.395
Women	25 (14.9)	143 (85.1)	
Men	4 (9.8)	37 (90.2)	
Age (years)			0.013
20-29	2 (4.3)	44 (95.7)	
30-39	9 (10.8)	74 (89.2)	
40-49	14 (23.0)	47 (77.0)	
50-59	2 (13.3)	13 (86.7)	
≥ 60	2 (50.0)	2 (50.0)	
Self-declared color/race			0.365
Black	2 (14.3)	12 (85.7)	
Brown	16 (12.2)	115 (87.8)	
Yellow	3 (33.3)	6 (66.7)	
White	8 (14.5)	47 (85.5)	
Profession			0.436
Physician	6 (10.5)	51 (89.5)	
Nurse	9 (12.3)	64 (87.7)	
Nursing Technician	14 (17.7)	65 (82.3)	
Work institution			-
Outpatient clinic	0 (0.0)	3 (100.0)	
Family health strategy	29 (13.9)	180 (86.1)	
Hospital	3 (16.7)	15 (83.3)	
Mobile emergency care service	0 (0.0)	3 (100.0)	
Time of work experience (years)			0.159
< 5	11 (12.5)	77 (87.5)	
5-10	6 (9.4)	58 (90.6)	
> 10	12 (21.1)	45 (78.9)	
Direct contact with			-
Perforating materials	28 (14.2)	169 (85.8)	
Minimally invasive procedures	12 (11.0)	97 (89.0)	
Surgical procedures	2 (16.7)	10 (83.3)	
Biological materials in laboratories	1 (14.3)	6 (85.7)	
HBV immunization (doses)			0.892
1	1 (16.7)	5 (83.3)	
2	2 (18.2)	9 (81.8)	
≥ 3	26 (13.5)	166 (86.5)	
Time of the last vaccine dose (years)			0.473
≤ 1	2 (8.7)	21 (91.3)	
2-5	12 (14.3)	72 (85.7)	
6-10	9 (16.4)	46 (83.6)	
11-15	1 (4.3)	22 (95.7)	
> 15	5 (20.8)	19 (79.2)	
Number of accidents with biological materials in the work environment			0.205
0	20 (13.7)	126 (86.3)	
1	5 (12.2)	36 (87.8)	
2	2 (11.1)	16 (88.9)	
≥ 3	2 (50.0)	2 (50.0)	
Previous knowledge of the anti-Hbs test			0.205
Yes, and already took it	17 (11.3)	134 (88.7)	
Yes, knew its importance, but did not take it	11 (20.4)	43 (79.6)	
Yes, but did not know its importance and did not take it	1 (100.0)	0 (0.0)	
No previous knowledge	0 (0.0)	3 (100.0)	

* Pearson’s chi-square test

Anti-Hbs = antibody against hepatitis B surface antigen; HBV = hepatitis B virus.

## DISCUSSION

Corroborating findings from other researchers,^14-16^ the sample in this study was predominantly composed of women health care workers, which reflects a characteristic of the health area in Brazil, in which women correspond to 78.9% of the workforce.^17^

In addition, most professionals declared themselves as brown, confirming the higher prevalence of brown people in Montes Claros, MG (59.04%), according to the last demographic census.^18^ Still on the sociodemographic data, the mean age of the sample was similar to a study conducted in Florianópolis, state of Santa Catarina (SC), among 1.249 health professionals, in which the mean age of all participants was 37.4 years (16-73 years; median 37),^19^ showing that, in both surveys, the population was mostly composed of young adults.

Regarding immunization, all health professionals included reported having taken at least one dose of the hepatitis B vaccine. This positive outcome reflects the results of the NIP policy that introduced free HBV vaccination for the Brazilian population in 2000, especially for health care professionals. The aim was to prevent infectious diseases related to occupational risk for workers and/or their patients.^11^

This also reflects the complete vaccination status of the sample, which was higher compared to other studies conducted on health professionals in Belo Horizonte, MG, (74.9%),^12^ Florianópolis, state of Santa Catarina (64.61%),^19^ Santo Antônio de Jesus, state of Bahia (BA) (59.9%),^20^ and Teresina, state of Piauí (PI) (51.1%).^14^

Low prevalence of HBV vaccination was also found in other countries, such as Ethiopia (37.7%),^21^ Sweden (40%),^22^ and Pakistan (40%).^23^ In these countries, the determinants of non-complete vaccination were mainly the high cost of the vaccine and the lack of information by the professionals. A Brazilian state that resembled the prevalence of these countries was the state of Pará (PA), which had only 31.6% of professionals fully vaccinated.^24^

In addition to having a full vaccination schedule, it is recommended that health care workers also confirm hepatitis B immunization through a serological test, because not all vaccinated individuals will seroconvert after taking the vaccine.

Also, antibody levels may decrease over the years,^3^ so in this study, non-reactive individuals are professionals who did not seroconvert and those who had anti-Hbs levels reduced to non-reactive results over the years. Therefore, high-risk groups should be monitored and advised to take another dose of the vaccine if the anti-Hbs titer reduces to levels ≤ 10 UI/mL (the equivalent of non-reactive).

Therefore, since healthcare professionals represent a population vulnerable to HBV infection, anti-HBV testing should be performed 30 to 60 days after the last dose of the vaccination scheme.^5^ In Brazil, the rate of performance of this test after primary vaccination in health care workers ranged from 32.9 to 61.7%,^19,20^ which was lower than that found in this study. Despite this high percentage, it is still important to raise awareness among health care workers about the importance of anti-Hbs testing in the context of public health to increase the prevalence of immunized individuals in Brazil.

In the present study, the rate of professionals who did not show seroconversion to the vaccine represented a higher value than that presented in an African study from 2019 (8.2%).^25^ However, a survey from BA had a similar result, with 13.4% of health workers not reagent to the vaccine.^20^ Similarly, a study conducted in the state of São Paulo (SP) showed 16.6% seronegativity among participants.^15^ In contrast, studies in other countries, such as India^26^ and Uganda,^16^ showed higher prevalence rates of 30.0 and 48.9%, respectively. These results are a consequence of a lack of information and poor vaccination coverage in these countries, negatively influencing the full immunization of health professionals.

In this context, although the results obtained were low compared to international studies, when considering that hepatitis B vaccination coverage and anti-Hbs tests are freely available for health professionals in Brazil,^5^ the non-seroconversion rate was significant. This corroborates the fact that, although there are prevention and control programs against viral hepatitis, there is a need for greater dissemination of the importance of serological confirmation after vaccination in the health field.

The response after vaccination, represented by the production of antibodies against hepatitis B, depends on the type, dose, and vaccination schedule used, as well as on age, sex, genetic factors, comorbidities, and immune system status of the vaccinee.^27^ However, in the present study, age was the only factor significantly associated with seroconversion and the number of vaccine doses taken.

The evidence reinforces a previous study,^28^ which showed that young adults have a higher seroprotection rate for the hepatitis B vaccine than other age groups. Furthermore, a full course of the hepatitis B vaccine induces protective antibody levels in more than 95% of adults. After age 40, this protection drops to less than 90%; and by age 60, protective antibody levels are achieved in only 65-75% of those vaccinated.^27^

Contrary to a survey conducted with professionals from the state of Piauí,^14^ the present study showed no statistically significant association between the variable number of vaccine doses and the professional category. The higher number of vaccine doses also failed to influence the positivity of the anti-Hbs test, as shown in a 2019 African study.^25^

Although a considerable number of the workers evaluated here stated that they had experienced accidents with biological material at least once in the work environment, no professional reported previous HBV infection. This fact is a positive consequence of national health promotion programs, which have contributed to significant changes in health-disease patterns in Brazil, contributing to the decline in morbidity and mortality from infectious diseases.^29^ This is contrary to a study from Tanzania, where the prevalence of hepatitis B among health care workers was 7%.^30^ The higher infection rates among health care workers in this country reflects the lack of public policies to control diseases preventable by immunization.

This study is not without limitations, such as the use of self-reported data, which may influence the reliability of the findings and likely recall bias. Another limitation was that only primary care health professionals were investigated. In addition, although cleaning and general services assistants are also a risk group, they were not included in the study because they were not included in the list of servers on the CNESNet, making it impossible to calculate the sample. Finally, since there was no access to the previous results of anti-Hbs tests performed by the participants 60 days after the third dose of vaccine, it was not possible to differentiate among the non-reactive population how many individuals did not seroconvert or had their antibodies reduced to non-reactive levels over the years.

## CONCLUSIONS

The results obtained are ratified by other studies previously conducted on the same main topic. Although the vast majority of health professionals tested reported having taken the three recommended doses of the HBV vaccine, the number of non-seroconversion was high.

The parameters evaluated in this study corroborate the epidemiological aspects of public health. Thus, the interpretation of its results, as well as of similar studies, should be taken into consideration for the planning of health actions for the studied population.

In the definition of policies related to immunization, the NIP, in its broad activity and notorious participation in the control of several infectious diseases, has the opportunity to collaborate for testing the immunity of the population. Such aspects can contribute to the possible eradication of the disease.
